# Comparative Evaluation of Two Bracket Systems’ Bond Strength: Conventional and Self-Ligating

**DOI:** 10.3390/dj10100196

**Published:** 2022-10-21

**Authors:** Aurel-Claudiu Vartolomei, Dana-Valentina Ghiga, Dan-Cosmin Serbanoiu, Marioara Moldovan, Stanca Cuc, Mariana Pacurar, Maria Cristina Figueiredo Pollmann

**Affiliations:** 1 Faculty of Dental Medicine, GEP University of Medicine Pharmacy, Science and Technology of Targu Mures, 540139 Targu Mures, Romania; 2 Raluca Ripan Chemistry Research Institute, Babes Bolyai University, 400294 Cluj-Napoca, Romania; 3 Faculty of Dental Medicine, University of Porto, 4200-393 Porto, Portugal

**Keywords:** shear bond strength, adhesion, active self-ligating brackets, passive self-ligating brackets, conventional brackets, tensile strength, maximum load, testing machine

## Abstract

Adhesion remains a key element in dentistry, whether approached in prosthetics, odontology, or orthodontics. It is a continuously researched aspect, as improved materials and adhesive methods keep emerging in the market. No orthodontic treatment can be effective without the proper adhesion strength of the bonded elements on the teeth. The objective of this research, in the broad context of self-ligating versus conventional brackets, was to compare active and passive self-ligating systems with a conventional one by conducting an in vitro study on human-extracted premolars. Shear bond strength tests were executed by means of an advanced materials-testing machine that generated maximum load and tensile strength values. The data obtained underwent statistical analysis with a statistical threshold of *p* < 0.05. The results regarding the statistical significance were acquired when comparing the passive self-ligating system with the active self-ligating and conventional systems (load-at-maximum-load mean 204.9, SD 91.09, and *p* < 0.05). In this study, the passive self-ligating bracket system appears to present increased shear bond strength.

## 1. Introduction

In the vast field of bracket features, adhesion remains an important aspect to be taken into consideration. Whether it is a characteristic that determines the choice of system remains the practitioner’s personal decision.

Adhesive interfaces have a critical impact on clinical success in dentistry and interface durability can be quantified by in vitro tests [1,2,3]. Shear bond strength testing is appropriate for testing orthodontic materials that are bonded to enamel. More than 1000 studies have been performed that analyze the different factors influencing the bond strength of orthodontic brackets [4].

Research has evaluated different bond strength variables including the surface (enamel, metal, and ceramic), adhesive system (glass ionomer and composite), bracket material (stainless steel), and type (self-ligating, conventional, and lingual), together with the type of bracket base examination, the placement force, the surface pretreatment, or the enamel’s contamination with saliva or blood [5,6,7,8,9,10].

Additionally, few clinical studies have been performed that assess this aspect using in vivo debonding devices and comparing the results with in vitro studies [11,12]. Furthermore, clinical bond failure is a multifactorial issue that must be taken into consideration when assessing adhesion. The related risk factors include the bracket material, placement site, type of anomaly (overjet, overbite, and dental classification), patient age, oral hygiene, and treatment phase [13,14].

There are two types of self-ligating brackets: active and passive. Active self-ligating brackets apply an active force on the archwire through a spring clip that maintains it in the slot. Passive self-ligating brackets have an additional slide that once locked does not affect the slot and does not express any active forces on the archwire, transforming the bracket into a tube [15,16]. Conventional brackets maintain the archwire by means of replaceable stainless steel or elastomeric ligatures. Self-ligating brackets are bulkier than conventional brackets and, together with the bracket base’s design, this can influence the adhesion to enamel [17,18].

In order to test the bond strength, usually, a shear force is applied in a testing machine with a certain crosshead speed until the failure of the adhesive system. The debonding force is registered in newtons/megapascals (interface stress units).

There are no clear guidelines in the literature regarding bond strength limits but the material should permit good adhesion to sustain masticatory forces (5–10 MPa minimum shear bond strength) [19] and not exert excessively strong adhesion forces that can lead to enamel loss when debonding (40–50 MPa) [20].

The purpose of this in vitro study was, thus, to compare active and passive self-ligating bracket systems with a conventional one with respect to shear bond strength. The null hypothesis is that there is no significant difference between their enamel adhesion properties.

## 2. Materials and Methods

A total of 84 stainless-steel maxillary first bicuspid brackets (n = 84) were bonded on 84 extracted teeth with the use of the following brackets: 28 conventional brackets, 28 active self-ligating brackets, and 28 passive self-ligating brackets. The systems utilized were Master Series (metallic conventional Roth 0.022-inch slot, American Orthodontics, Sheboygan, WI, USA), Empower 2 Interactive (active), and passive self-ligating brackets (metallic Roth 0.022-inch slot, American Orthodontics, Sheboygan, WI, USA).

**Figure 1:**
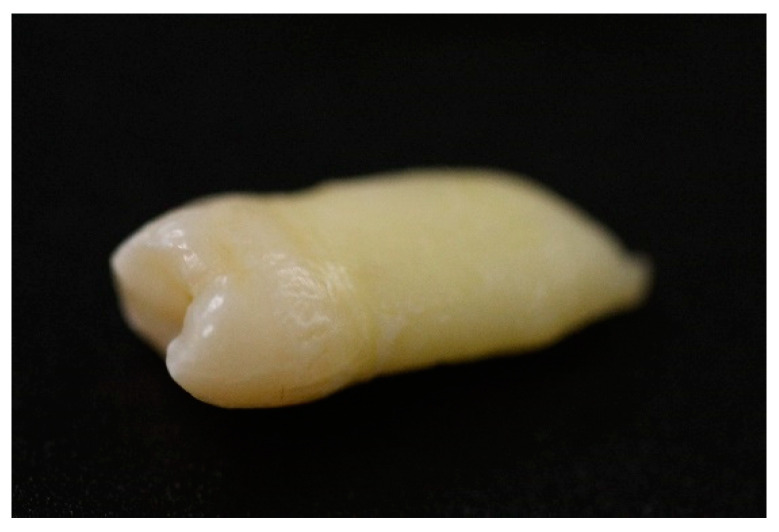
Extracted premolar.

The extracted teeth were acquired from the oral and maxillofacial surgery department of the GEP University of Medicine, Pharmacy, Science, and Technology of Targu Mures with the approval of the Ethics Committee (No. 1779 from 10.06.2022). Only teeth with intact enamel and proper anatomical buccal surfaces for suitable bicuspid bracket bonding were included. A sample tooth can be seen in Figure 1.

Teeth with fillings, scale, cavities, large white spot lesions, cracks on the buccal sides, or less than two thirds of the roots present were excluded [21]. They were thoroughly cleaned with saline solution after all organic material had been removed and preserved in artificial saliva after extraction. Artificial saliva composition can be seen in Table 1. It comprised water, hydrochloric acid, calcium chloride, sodium bicarbonate, and disodium phosphate.

**Figure 2:**
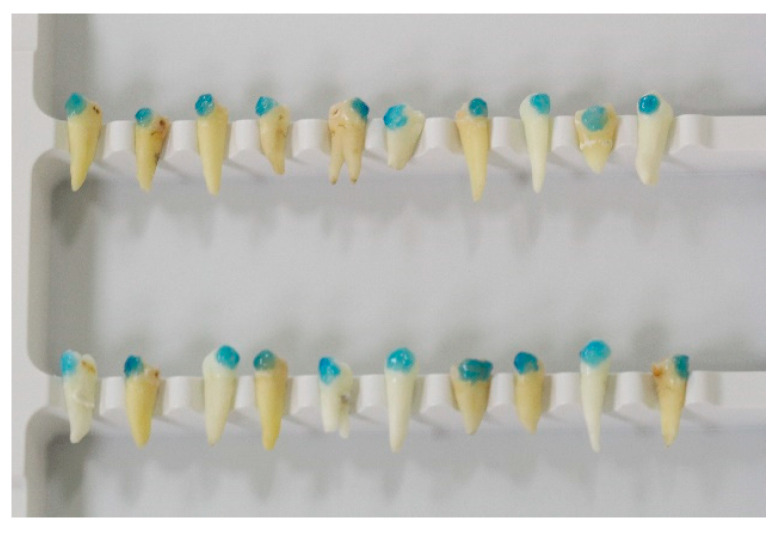
Etched premolars.

**Figure 3:**
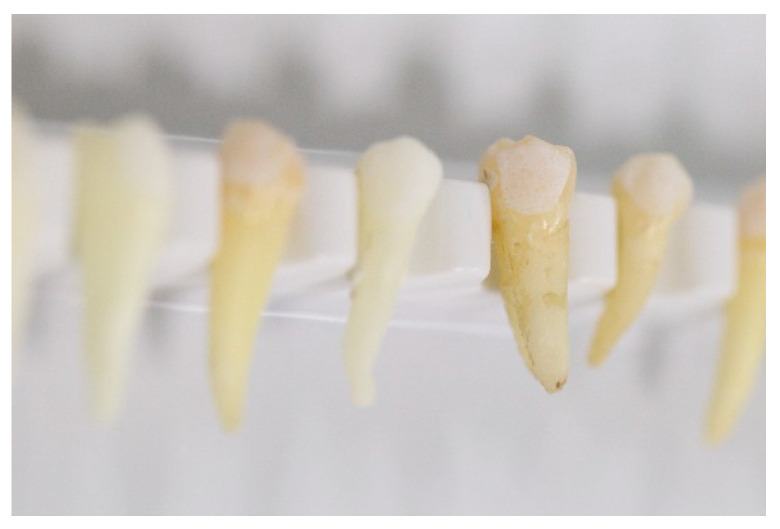
Rinsed and dried premolars.

The teeth were brushed without toothpaste, etched with Blue Etch (36% orthophosphoric acid, Cerkamed, Stalowa Wola, Poland) for 20 s, rinsed, and dried (notice Figure 2 and Figure 3). The brackets were bonded using Orthosolo Universal Bond Enhancer, Ormco, and Transbond XT composite, 3M. They were light-cured separately with Woodpecker iLed for 3 s after the excess material was removed.

**Figure 4:**
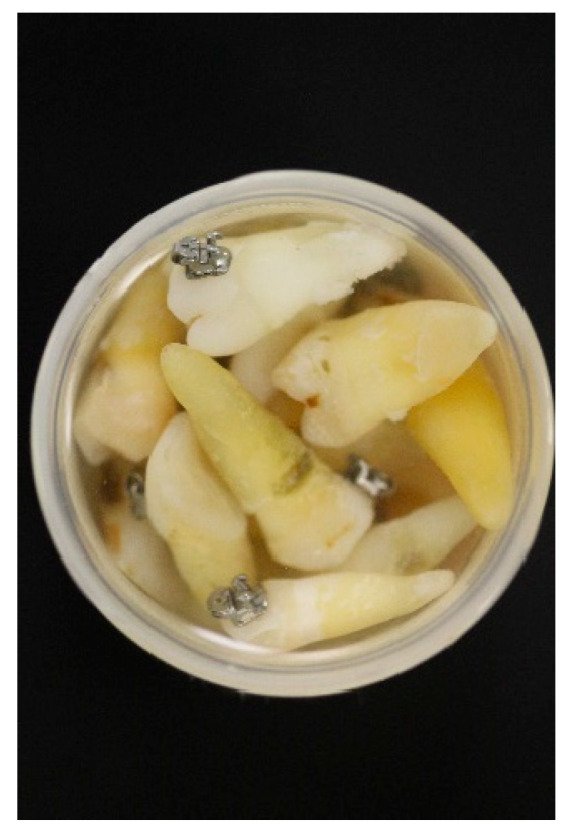
Teeth with bonded brackets in artificial saliva.

For 1 month, the teeth were conserved again in artificial saliva until the measurements were performed (as can be seen in Figure 4).

The vast majority of studies have been performed using brackets bonded to bovine teeth [22,23,24]. Although bovine enamel has been described to be a reliable replacement for human enamel in bonding research, this study was conducted using human premolar teeth for better accuracy [22]. Furthermore, the teeth were preserved in artificial saliva to better simulate the intra-oral environment [25].

We could not identify any studies in the literature that compared active and passive self-ligating brackets regarding bonding strength nor any study that focused solely on the brackets utilized in this research.

Bond strength was measured by applying an American Society for Testing and Materials D638 compression test of a Lloyd type LR5K Plus Dual-Column mechanical testing machine (Ametek, Lloyd Instruments, Meerbusch, Germany), with a 5 KN maximal approved capacity. The device is equipped with an electronic compression force measurement and transmission structure. For reproducibility, the selected basic settings can be seen in Table 2.

Data processing was performed in NexygenPlus software.

**Figure 5:**
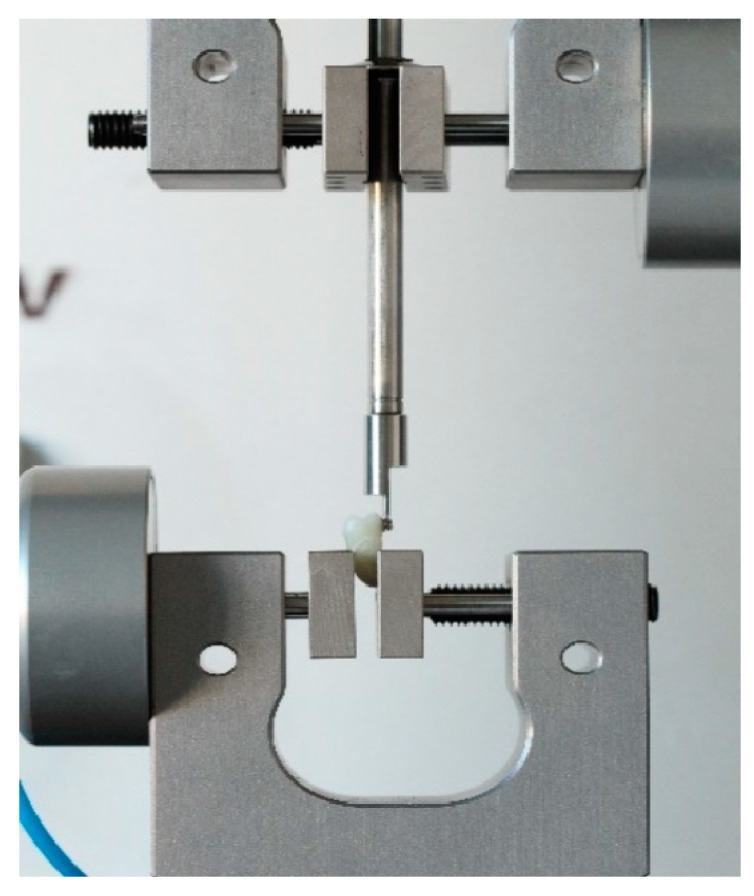
Setup for the test.

**Figure 6:**
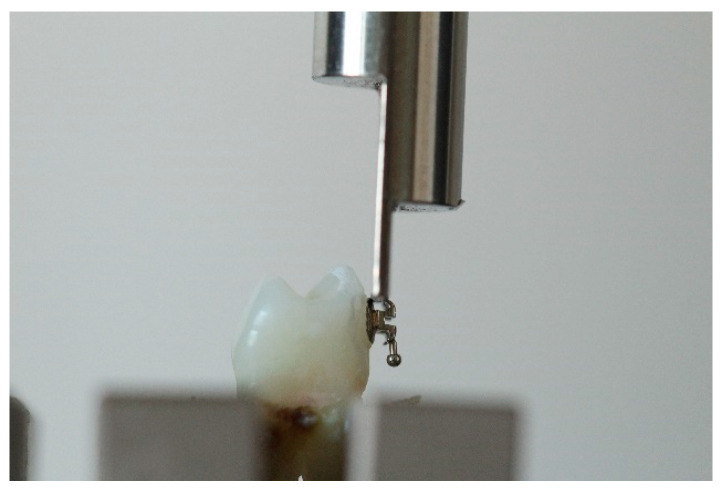
Pin pressing on conventional bracket.

Each tooth with the bonded bracket was firmly placed in a load cell that was locked into the lower hydraulic grip in a fixed, repeatable position. The upper hydraulic grip with a metal pin in place would perpendicularly press against the bracket between the wings and the base at the selected crosshead speed, as can be seen in Figure 5 and Figure 6 [26,27].

All actions and measurements were performed by the same operator.

The maximum stress a material can tolerate when being pulled or stretched before breakage represents its tensile strength. It is calculated by executing a tensile test and registering the stress–strain curve, wherein the uppermost point of is the tensile strength [28,29]. It is quantified as force per area unit. The unit is the Pa, pascal, or a multiple, MPa, megapascal. Equivalently, it is measured in N/m^2^—newtons per square meter [30].

The maximum load is the load limit that a body can withstand safely. It is the load at which the structure is in a state of initial plastic collapse. Plasticity spreads throughout the structure and at the maximum load, the plastic zone becomes large, and the component is considered to have collapsed. In physics, it is measured in N, newtons [31].

The computer displayed the stress–strain graphic and the load at maximum load and tensile strength values. A total of 84 measurements were performed, one for each bracket-tooth couple: twenty-eight for the conventional brackets (CB), 28 for the active self-ligating brackets (ASLB), and 28 for the passive self-ligating brackets (PSLB). The values obtained were compared as follows:

1. ASLB vs. PSLB;
2. ASLB with CB;
3. PSLB with CB.

The statistical analysis included inferential statistics and descriptive statistics elements: mean, median, and standard deviation. The Shapiro–Wilk test was applied to determine the analyzed data series distribution. For non-normal distributions, for median comparison, the Kruskal–Wallis test and Dunn’s Multiple Comparison Test as a post hoc test were performed. We used Univariate Analysis of Variance to calculate the post hoc Power of the sample size. The significance level for the *p*-value was 0.05. The statistical analysis was accomplished in the demo version of GraphPad Prism utility program [32].

## 3. Results

Table 3 comprises the means, medians, and standard deviations of each of the three comparison groups for both the tensile strength and load-at-maximum-load measured values. The detailed statistical tables, as well the crosshead speed tables with all the performed measurements, are accessible on request.

Using the Univariate Analysis of Variance, for tensile strength, the post hoc power of a sample size with 28 subjects in each group is 15.8%, meaning there is a 15.8% chance to detect a difference between the values in the three groups.

For the load at maximum load, the post hoc power of a sample size with 28 subjects in each group is 72.4%, meaning there is a 72.4% chance to detect a difference between the values in the three groups [33].

The Kruskal–Wallis test showed that there is no statistically significant difference between the medians of the tensile strength values in ASLB, PSLB, and CB (*p* > 0.05).

The Kruskal–Wallis test also showed that there is a statistically significant difference between the medians of the load-at-maximum-load values in ASLB, PSLB, and CB. Clinically, this can indicate an increased bond strength in the passive system (*p* < 0.05).

The results of Dunn’s Multiple comparison post hoc test are given in Table 4.

Statistically significant differences were found when comparing the passive self-ligating system with the active self-ligating system and the conventional system.

## 4. Discussion

Naturally, the adhesive system is of paramount importance when discussing this topic. In a comparative four-adhesive study, Transbond XT attained the highest bonding strength [34]. The same result was obtained when comparing two different bracket bases [8].

The existing research mainly focuses on different types of materials when comparing the bracket shear bond strengths. Ceramic brackets present a significantly lower bond strength when compared to stainless-steel brackets [35]. Another study concluded that ceramics composed primarily of zirconia should be treated with at least three coats of methacryloyloxy-decyl dihydrogen phosphate primer in order to manifest a bond strength similar to that of a silica-rich ceramic treated with hydrofluoric acid and silane. The study was performed on orthodontic tubes [36].

Bonding orthodontic brackets on zirconia restorations is still a clinical challenge. Pretreatment with silica-coated alumina and applying RelyX or Clearfill ceramic primer provided sufficiently high shear bond strength values [37].

Increasing brackets’ shear bond strength is of great of interest. Enamel surface deproteinization with sodium hypochlorite before etching increased the bond strength of glass ionomer cement to similar values of Transbond XT, potentially reducing the incidence of white spot lesions [38]. Pretreating enamel with an Er,Cr:YSGG laser and CPP-ACP as a preventive measure before bonding brackets does not reduce bond strength [26]. Still, Er:YAG laser irradiation does not represent an option for enamel conditioning according to Contreras-Bulnes et al. [39]

Phosphoric acid produced a more aggressive etching pattern when compared to polyacrylic acid and self-etching primers, but the shear bond strength was not significantly influenced by the adhesive system or etching pattern [40]. Enamel deproteinization with sodium hypochlorite enhanced the bond strength in metal brackets but there was no statistically significant difference among the groups treated or untreated with this substance [41].

Fluoride pretreatment applied on eroded enamel before bracket bonding leads to a decrease in shear bond strength, while a fluoride-releasing adhesive can increase bonding to eroded surfaces [42]. In addition, the pretreatment of enamel with erythritol could represent a feasible method to reduce the failure rate in brackets [43].

In another study, airborne particle abrasion with aluminum trioxide and silica coating followed by silanization provided the highest bond strength when bonding polycarbonate brackets to ceramic [44]. Glass ionomer cement enriched with nano-titanium particles is a promising material due to its durability with respect to withstanding mastication forces and potential antibacterial activity [9]. Applying the OrthoPrimer bonding agent instead of XT Primer (3M) provided a higher bond strength of sandblasted polycarbonate bracket bases to enamel, thus making debonding more difficult and causing fractures of the bracket wings [45]. Exerting a sustained sealing force when bonding orthodontic brackets improves the adhesive layer’s quality and increases bond strength but does not influence the adhesive remanent index score distribution [6]. The combination of hydro-abrasion and acid etching significantly increased the shear bond strength values in orthodontic brackets [46].

In one study, an in-office reconditioning procedure of self-ligating brackets altered the shear bond strength but its values remained clinically acceptable [47].

Similar studies found increased shear bond strength values in self-ligating brackets when compared to conventional or other self-ligating brackets [7]. Northrup et al. conducted another study comparing two adhesives with conventional and self-ligating brackets and concluded that the Damon self-ligating system presented satisfactory shear bond strength with both adhesives (Transbond and Blugoo) [48]. Basaran and Ozer also found a clinically acceptable mean bond strength among two self-ligating systems and a conventional one but with no significant differences [33]. Sfondrini et al. arrived at similar outcomes [4].

In vitro study-associated limitations are to be taken into consideration when discussing the findings of this research. It is best that the results of this study and of similar studies be confirmed by clinical trials. A linear experiment cannot mimic the dynamic complex interactions that occur in the intra-oral three-dimensional environment. Furthermore, the operation of the LR5K machine was performed manually, which can lead to some degree of testing error. The lack of homogeneity in the methodology applied in multiple studies results in certain reservations regarding the interpretation of the comparative results. The interactive (active) and passive bracket systems from American Orthodontics present the same base design. The identification of statistically significant differences between the two in our research generated discussions on their source. As the results are linked to the tested materials, the resilience of the closing mechanism can represent a factor as the debonding pressure was applied at said mechanism’s level. Other bracket systems may present stiffer clips, but further studies should be conducted regarding this aspect. Is this of absolute importance if the bond strength is acceptable for finishing an orthodontic treatment? None of the methods described above have been applied to enhance enamel adhesion.

Thus, the null hypothesis is rejected.

## 5. Conclusions

Passive self-ligating brackets presented the highest shear bond strength, but all three systems presented suitable adhesion strength. Future clinical trials are required to support the findings of this research.

## Figures and Tables

**Table 1:** Percentage components of artificial saliva.

Composition	%
Na_2_HPO_4_	0.3
NaHCO_3_	
CaCl_2_	
HCl-1M	0.3
H_2_O	99.4

**Table 2:** Testing machine settings.

Direction	Tension
Preload/stress	0.5 N
Preload/stress Speed	1 mm/min
Automatically zero at the start of the test	Load extension
Test speed–Extension rate	1 mm/min

**Table 3:** Mean, Median and SD.

	ASLB (n = 28)	PSLB (n = 28)	CB (n = 28)	*p*-Value
Tensile Strength			
Median	10.84	13.94	11.07	0.3474
Mean	11.89	14.03	13.35	
Std. Deviation	7.085	6.370	7.750	
	Load at Maximum Load			
Median	139.3	205.8	116.6	0.0114
Mean	153.0	204.9	140.7	
Std. Deviation	90.81	91.09	81.66	

**Table 4:** Dunn’s Multiple Comparison Test.

	Dunn’s Multiple Comparison Test *p*-Value
Tensile Strength	
ASLB versus PSLB	0.1425
ASLB versus CB	0.5830
PSLB versus CB	0.4173
Load at Maximum Load	
ASLB versus PSLB	0.0373
ASLB versus CB	0.5830
PSLB versus CB	0.0052

## Data Availability

Not applicable.
